# Use of zebrafish to study *Shigella* infection

**DOI:** 10.1242/dmm.032151

**Published:** 2018-02-01

**Authors:** Gina M. Duggan, Serge Mostowy

**Affiliations:** Section of Microbiology, MRC Centre for Molecular Bacteriology and Infection, Imperial College London, London SW7 2AZ, UK

**Keywords:** Antimicrobial resistance, Autophagy, Cytoskeleton, Emergency granulopoiesis, Inflammation, Macrophage, Neutrophil, Septin, *Shigella*, Zebrafish

## Abstract

*Shigella* is a leading cause of dysentery worldwide, responsible for up to 165 million cases of shigellosis each year. *Shigella* is also recognised as an exceptional model pathogen to study key issues in cell biology and innate immunity. Several infection models have been useful to explore *Shigella* biology; however, we still lack information regarding the events taking place during the *Shigella* infection process *in vivo*. Here, we discuss a selection of mechanistic insights recently gained from studying *Shigella* infection of zebrafish (*Danio rerio*), with a focus on cytoskeleton rearrangements and cellular immunity. We also discuss how infection of zebrafish can be used to investigate new concepts underlying infection control, including emergency granulopoiesis and the use of predatory bacteria to combat antimicrobial resistance. Collectively, these insights illustrate how *Shigella* infection of zebrafish can provide fundamental advances in our understanding of bacterial pathogenesis and vertebrate host defence. This information should also provide vital clues for the discovery of new therapeutic strategies against infectious disease in humans.

## Abstract (precis)

**Summary:** Here, we review how studying *Shigella* infection of zebrafish has illuminated novel research avenues in both infection and cell biology.

## Introduction

*Shigella* are a pathovar (see Glossary, Box 1) of *Escherichia coli* that cause dysentery (see Glossary, Box 1) via inflammatory destruction of the intestinal epithelium, a disease process called shigellosis. Up to 165 million cases of shigellosis are estimated to occur annually, resulting in up to half a million deaths (Lima et al., 2015; Kotloff et al., 2017). Moreover, *Shigella* infection can give rise to serious postinfectious sequelae (see Glossary, Box 1), such as arthritis, sepsis, seizures and haemolytic uremic syndrome (see Glossary, Box 1). Similar to other Gram-negative (see Glossary, Box 1) pathogens, cases of *Shigella* with acquired resistance to fluoroquinolones (see Glossary, Box 1) and other antibiotics are rising (Harrington, 2015), and the World Health Organization (WHO) has listed *Shigella* among its top 12 priority pathogens requiring urgent action (WHO report, 2017). Among the four *Shigella* subgroups, *Shigella flexneri* is the most common cause of dysentery in low-income countries and the most prevalent of the *Shigella* subgroups in children under 5 years of age (Connor et al., 2015). By contrast, infection from *Shigella sonnei* predominates in developed countries (Kotloff et al., 1999; Holt et al., 2012; Kotloff et al., 2017). The infection process of *S. sonnei* remains poorly understood compared to that of *S. flexneri*, and therefore the majority of our current knowledge is extrapolated from work performed using *S. flexneri*. Important differences between subgroups have been described genetically, but have not been fully tested in infection models, for example the presence of a chromosomally encoded type VI secretion system (T6SS; see Glossary, Box 1) in *S. sonnei*, which is absent in *S. flexneri*.

**Box 1. Glossary**

**Actin tails:** Propulsive tails that result from the polymerisation of the host cell actin by intracytosolic pathogens to aid them in disseminating from cell-to-cell.

**ASC speck:** A platform for caspase-1 activity and readout for inflammasome activation.

**Autophagosome:** Double-membraned vesicle that compartmentalises cellular material targeted to autophagy.

**Autophagy:** A highly coordinated process of intracellular degradation whereby cytosolic components are isolated within a double-membrane vacuole (autophagosome) and targeted for lysosomal destruction.

***Bdellovibrio bacteriovorus*:** A Gram-negative bacterium that parasitises other Gram-negative bacteria by invading their periplasmic space, undergoing replication, and killing their prey.

**Caspase:** Cysteine protease controlling inflammation and programmed cell death.

**Cell-autonomous immunity:** The ability of a host cell to independently eliminate infectious agents using antimicrobial defences and host cell death.

**CRISPR/Cas9:** A genome editing approach adapted from the antibacteriophage defence system discovered in bacteria.

**Cytokinetic furrow:** A micron-scale invagination of the cellular surface during cytokinesis, leading to cell division.

**Dysentery:** Gastroenteritis resulting in bloody diarrhoea.

**E3 ubiquitin ligase:** Ligating (E3) enzymes that, together with ubiquitin activating (E1) and conjugating (E2) enzymes, mediate ubiquitylation (a post-translational modification of proteins).

**Emergency granulopoiesis:** *De novo* generation of neutrophils that arise from increased myeloid progenitor cell proliferation in response to infection and leukocyte exhaustion.

**Haemolytic uremic syndrome:** A life-threatening condition caused by the destruction of red blood cells.

**Fluoroquinolones:** A family of broad spectrum antibacterial agents used in human and veterinary medicine.

**Gram-negative bacteria:** A group of bacteria that lose the Crystal Violet dye in the Gram's method of staining owing to the structure of their cell wall.

**Guanylate-binding proteins:** A family of GTPases induced by IFNγ, and key components of cellular immunity.

**Haematopoietic stem and progenitor cells:** Cells that proliferate (into haematopoietic stem cells) and differentiate (into neutrophils) to mediate emergency granulopoiesis.

**Inflammasome:** A multi-protein complex and component of the innate immune system that promotes the maturation of inflammatory cytokines through recruitment of Caspase-1.

**Interferon regulatory factor 8:** A transcription factor required for lineage commitment and myelopoiesis.

**Larva:** The juvenile form zebrafish undergo before developing into adults.

**Macropinocytosis:** A nonselective, actin-dependent mechanism of cellular uptake whereby plasma membrane protrusions fold inwards to form vesicles (termed macropinosomes).

**Mammalian target of rapamycin:** A highly conserved kinase used by cells for nutrient sensing.

**Metronidazole:** A pro-drug used on transgenic fish (engineered to express nitroreductase using a cell/tissue-specific promoter) to ablate specific cells/tissues.

**Morphant:** An organism which has been genetically manipulated using morpholino oligonucleotides.

**Morpholino oligonucleotide:** ∼25 base nucleic acid analogues that affect RNA maturation or translation by sequence-specific base-pairing.

***Mycobacterium leprae*:** A species of bacteria that is the causative agent of leprosy in humans.

***Mycobacterium tuberculosis*:** A species of bacteria that is the causative agent of tuberculosis in humans.

**Neural Wiskott-Aldrich syndrome protein:** Induces actin polymerisation through the actin-related protein (Arp2/3) complex; it is a specific ligand for *Shigella* IcsA.

**Neutropenia:** A condition in which neutrophil number is decreased.

**Neutrophil extracellular traps:** Net-like structures comprised largely of decondensed chromatin that are released by neutrophils at sites of acute or chronic inflammation.

**Nonmuscle myosin II:** Actin-binding protein with contractile properties.

**Orthologues:** Genes in different species that evolved from a common ancestral gene.

**Paralogues:** Two or more genes that derive from the same ancestral gene, originating via genetic duplication.

**Pathovar:** Bacterial strains with similar characteristics.

**Peptidoglycan:** A polymer of amino acids and sugars that comprises the bacterial cell wall.

**Phagocytic cup:** A micron-scale cup-shaped invagination of the cell membrane formed during phagocytosis.

**Phagocytosis:** A process by which cells engulf particles, including bacterial pathogens.

**Prostaglandin D2:** A type of lipid signalling molecule produced at sites of tissue damage or infection to control inflammation.

**Pyroptosis:** A highly inflammatory form of programmed cell death that can occur in response to the presence of intracellular bacteria.

***Salmonella enterica* serovar Typhimurium:** A zoonotic (transmitted from animals) pathogen that causes gastroenteritis and inflammation of the intestinal mucosa.

**Septins:** A highly conserved family of GTP binding proteins that interact with the membrane and actin to form higher-order structures including filaments, rings and cages.

**Sequelae:** Chronic conditions resulting from infection or injury.

**Tumour** **necrosis** **factor:** A monocyte-derived pleiotropic pro-inflammatory cytokine involved in a spectrum of biological processes, such as induction of apoptosis.

**Type III secretion system:** A membrane embedded needle-like structure present in Gram-negative bacteria used to inject effector proteins into a host cell.

**Type VI secretion system:** A contractile nanomachine used by Gram-negative bacteria to puncture target cells and deliver effectors.

In addition to being an urgent health threat, *S. flexneri* is recognised as a paradigm for the investigation of cell biology and innate immunity (Picking and Picking, 2016). Through decades of work performed *in vitro* using the infection of cultured cells, *Shigella* has been a valuable model for dissecting how bacteria can invade nonphagocytic cell types (Cossart and Sansonetti, 2004; Haglund and Welch, 2011), form actin tails (see Glossary, Box 1) for cell-to-cell spread (Haglund and Welch, 2011; Welch and Way, 2013), and be recognised by cellular immunity (see Glossary, Box 1) for host defence (Phalipon and Sansonetti, 2007; Ashida et al., 2011, 2015). In an effort to fully decipher the molecular and cellular mechanisms underlying the *Shigella* infection process, the field is progressively shifting towards *in vivo* investigation using relevant animal models.

### The zebrafish infection model

To date, our capability of understanding the *Shigella* infection process *in vivo* has been limited. Although no nonprimate animal model exists that closely mimics shigellosis in humans, a variety of steps underlying the *Shigella* infection process can be examined using the rabbit (Arm et al., 1965; Perdomo et al., 1994; Schnupf and Sansonetti, 2012), guinea pig (Sereny, 1955; Shim et al., 2007) and mouse models (Yang et al., 2014; Li et al., 2017). Although these mammalian models have provided significant advances in testing mechanisms underlying *Shigella* pathogenesis, they remain poorly suited for *in vivo* imaging of the cell biology of *Shigella* infection.

There are many advantages to using zebrafish larvae (see Glossary, Box 1) to study infection, including their rapid development, fully annotated genome (which is highly homologous to that of humans) and optical accessibility for noninvasive real-time imaging (Lieschke and Currie, 2007; Sullivan et al., 2017). Importantly, zebrafish larvae lack an adaptive immune system during early embryonic development, and thus allow specific study of innate immunity without cross-interference from the adaptive immune system (Lieschke and Trede, 2009). The zebrafish model is also genetically tractable, and therefore amenable to the generation of fluorescent transgenic lines and to targeted gene manipulation. In the case of transient depletion, gene manipulation can be achieved using morpholino oligonucleotides (see Glossary, Box 1; Li et al., 2016). However, morpholinos can elicit off-target effects, and alternative strategies might be required to validate the conclusions. In the case of stable genome editing, mutants can be efficiently generated using zinc finger nucleases (ZFNs) (Foley et al., 2009), transcription activator-like effector nucleases (TALENs) (Bedell et al., 2012), or clustered regulatory interspaced short palindromic repeats/CRISPR-associated protein 9 (CRISPR/Cas9; see Glossary, Box 1; Blum et al., 2015). These systems all induce a site-specific double-stranded break, repaired using a nonhomologous end joining mechanism (Ata et al., 2016). The CRISPR/Cas9 system is currently the most prevalent approach to generate zebrafish mutants.

**Fig. 1.**
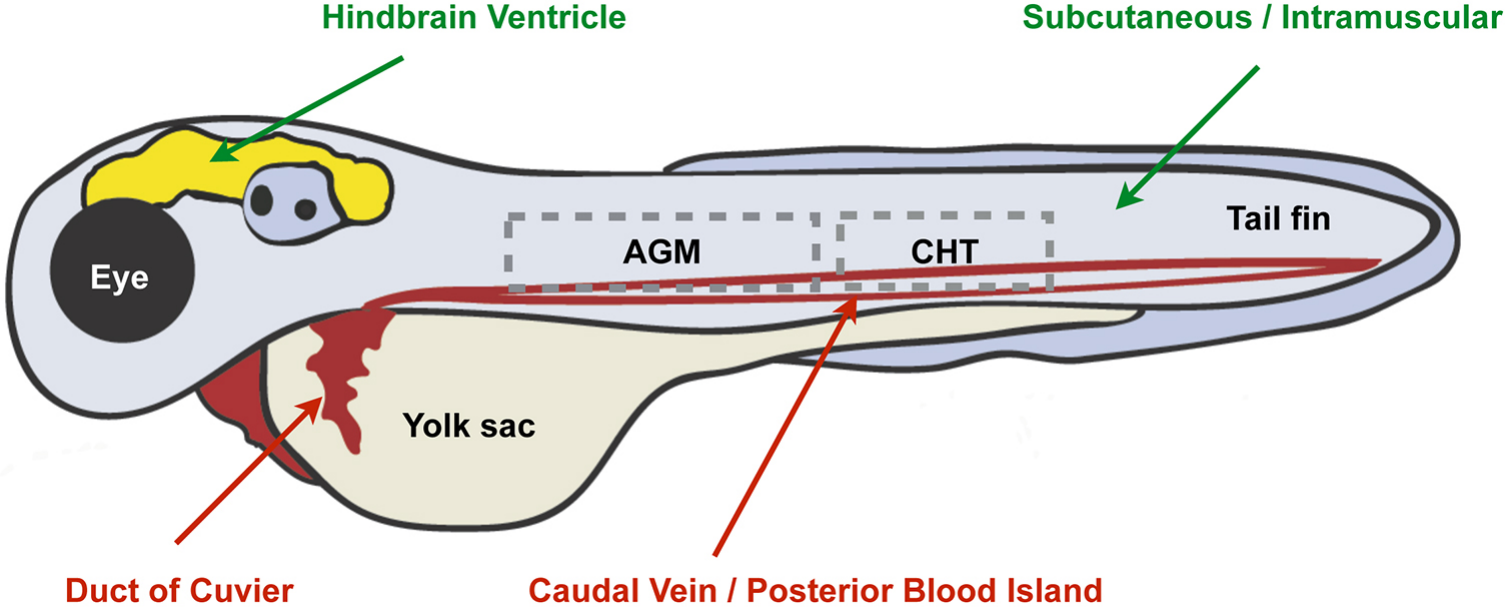
**Different injection sites of zebrafish larvae used to study *Shigella* infection.** Main attributes of different injection sites used for the study of *Shigella* infection of zebrafish larvae (3 days postfertilisation). To study systemic infection and *Shigella*-phagocyte interactions, intravenous injection of *Shigella* into the circulation is performed via the caudal vein/posterior blood island or Duct of Cuvier (highlighted in red). To study compartmentalised infection and a directed leukocyte response to *Shigella*, injection of *Shigella* into the hindbrain ventricle, or subcutaneous/intramuscular injection of *Shigella* into epithelial cells of the tail muscle, is used (highlighted in green). The dashed line boxes indicate the aorta-gonad-mesonephros (AGM) and caudal hematopoietic tissue (CHT) where emergency granulopoiesis (see Glossary, Box 1) takes place.

In recent years, our understanding of the infection process has expanded considerably through the use of the zebrafish infection model (reviewed in Renshaw and Trede, 2012). Originally used to study *Mycobacterium marinum*, a natural fish pathogen closely related to *Mycobacterium tuberculosis* (see Glossary, Box 1), zebrafish larvae have since been exploited to study pathogenesis and infection biology using a wide variety of bacteria (Masud et al., 2017; Torraca and Mostowy, 2017), viruses (Levraud et al., 2014; Varela et al., 2017) and fungi (Gratacap and Wheeler, 2014; Yoshida et al., 2017). For this purpose, injection of bacteria in the caudal vein/posterior blood island or Duct of Cuvier has been used to investigate systemic infection responses (Fig. 1), whereas injection in the tail muscle or hindbrain ventricle (HBV) has been used to analyse a directed leukocyte response to a compartmentalised infection (Fig. 1). Zebrafish infection models have been developed to study a variety of enteropathogens. For example, injection of *Salmonella enterica* serovar Typhimurium (see Glossary, Box 1) into zebrafish has been key for discovery of novel concepts in cellular immunity, immunometabolism, and emergency granulopoiesis (see Glossary, Box 1; reviewed in Torraca and Mostowy, 2017). Recent work has established zebrafish as a model for foodborne enterohaemorrhagic *E. coli* (EHEC) infection (Stones et al., 2017), a major cause of diarrhoeal illness in humans. Using the protozoan *Paramecium caudatum* as a vehicle for EHEC delivery, work has shown that zebrafish larvae can be used to study the hallmarks of human EHEC infection, including EHEC-phagocyte interactions in the gut and bacterial transmission to naive hosts (Stones et al., 2017). In the case of *Shigella*, caudal vein infection of zebrafish was first developed to study *Shigella*-phagocyte interactions and bacterial autophagy (see Glossary, Box 1) *in vivo* (Mostowy et al., 2013). Strikingly, many hallmarks of shigellosis observed in humans, including epithelial cell invasion, macrophage cell death and inflammation, are reproduced in a zebrafish model of *S. flexneri* infection, and are strictly dependent upon the *Shigella* type III secretion system (T3SS; see Glossary, Box 1). Moreover, studies using this model discovered a scavenger role for neutrophils in eliminating infected macrophages and other cell types that fail to control *Shigella* infection (Mostowy et al., 2013). In this case, when infected macrophages and other cell types fail to control infection, scavenger neutrophils act as a compensatory mechanism to clear both the dead macrophages and the infection.

Collectively, these reports introduced the zebrafish as a novel animal model to study the cell biology and innate immune response to *Shigella* at the molecular, cellular and whole-animal levels. In this Review, we highlight the diverse applications of *Shigella*-zebrafish infection, discussing the progress and insights achieved to date. We also discuss several open questions and future prospects.

## Recent mechanistic insights into *Shigella* infection

Here, we summarise a selection of mechanistic insights recently gained from studying *Shigella* infection of zebrafish. We focus on examples from two main themes: cytoskeleton rearrangements during infection, a historically important field of study critical for our understanding of host-pathogen interactions, and cellular immunity, a rapidly evolving field important for understanding host defence. These new mechanistic insights significantly expand our knowledge of the host response to *Shigella* infection, and also shed light on the general mechanisms crucial for host defence against bacterial pathogens.

### Cytoskeleton rearrangements during *Shigella* infection

Investigation of the cytoskeleton during bacterial infection has enabled major discoveries in both infection and cell biology (Cossart and Sansonetti, 2004; Haglund and Welch, 2011; Welch and Way, 2013). For example, how pathogens manipulate the host cytoskeleton to gain entry into cells and polymerise actin tails has revolutionised our understanding of phagocytosis (see Glossary, Box 1) and cell motility (Cossart and Sansonetti, 2004; Haglund and Welch, 2011; Welch and Way, 2013). In this section, we review the host cell response to *Shigella* invasion and actin-based motility, and discuss what has recently been learned from investigation using zebrafish.

**Fig. 2.**
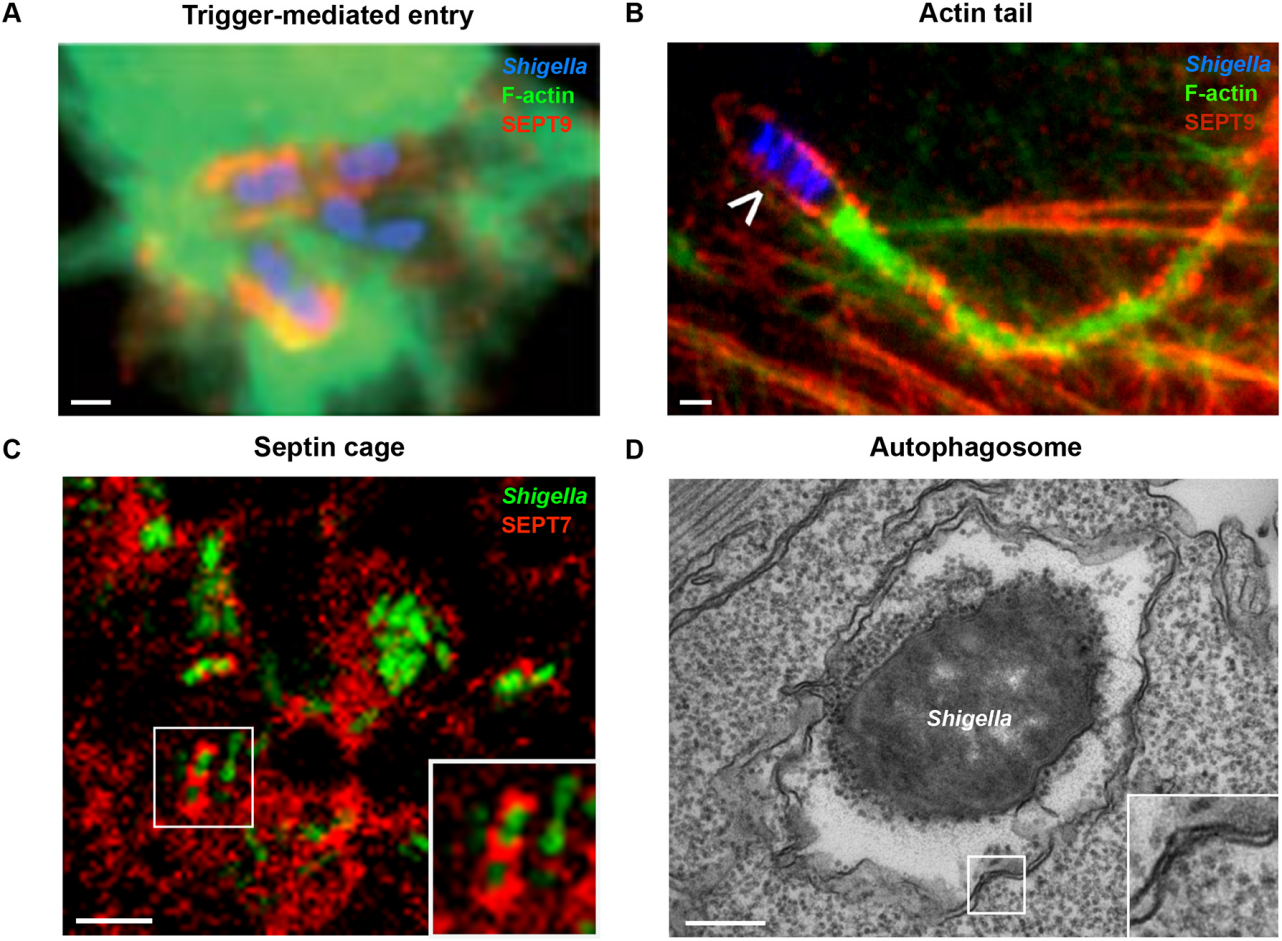
**The hallmarks of** ***Shigella*** **infection.** (A) Trigger-mediated entry by *Shigella*. HeLa cells were infected with *Shigella* (blue), fixed for fluorescent microscopy, and labelled with antibodies to SEPT9 (red) and phalloidin for F-actin (green) to highlight septin recruitment at the site of *Shigella* entry*.* Scale bar: 1 µm. (B) The *Shigella* actin tail. HeLa cells were infected with *Shigella* (blue; white arrowhead indicates a motile bacterium) for 3 h, fixed for fluorescent microscopy, and labelled with antibodies to SEPT2 (red) and phalloidin for F-actin (green) to highlight septin ring formation around the actin tails. Scale bar: 1 µm. (C) The *Shigella*-septin cage *in vivo*. SEPT7 (red) assembles into cage-like structures around *S. flexneri* (green)*.* Zebrafish larvae were infected with green fluorescent protein (GFP)-*Shigella* for 4 h, fixed, labelled with antibodies to SEPT7 and imaged by confocal microscopy. The inset shows a higher magnification view of the boxed region in C, showing *Shigella* entrapped within a septin cage. Scale bar: 5 µm. (D) An autophagosome sequestering cytosolic *Shigella in vivo.* Zebrafish larvae were infected in the tail muscle with GFP-*Shigella* for 4 h and fixed for electron microscopy. The inset shows a higher magnification view of the boxed region in D, showing the double membrane, a hallmark of autophagosomes. Scale bar: 0.25 µm. Images adapted from Mostowy and Cossart (2009) (A), Mostowy et al. (2010) (B) and Mostowy et al. (2013) (C,D).

*Shigella* uptake into nonphagocytic cells has been well characterised *in vitro*, where entry is dependent upon injection of T3SS effector proteins into the host cell (reviewed in Cossart and Sansonetti, 2004). This form of bacterial uptake, called trigger-mediated entry, causes the reorganisation of the host cell cytoskeleton via actin remodelling and plasma membrane ruffling (Fig. 2A). Engulfment occurs by a process analogous to macropinocytosis (see Glossary, Box 1), and, in the case of *Shigella*, is followed by vacuolar rupture and escape of bacteria into the cytosol (Weiner et al., 2016). Once in the cytosol, *Shigella* can recruit neural Wiskott-Aldrich syndrome protein (N-WASp; see Glossary, Box 1) to polymerise actin tails for its own motility (Fig. 2B), a process dependent on the bacterial outer membrane autotransporter IcsA (Goldberg and Theriot, 1995). Studies using human epithelial cells have discovered key roles for septins (see Glossary, Box 1) in the regulation of actin-mediated infection processes (reviewed in Torraca and Mostowy, 2016). A relatively poorly understood component of the cytoskeleton compared to actin, septins are important for a variety of cellular processes including cytokinesis and host-pathogen interactions (reviewed in Saarikangas and Barral, 2011; Mostowy and Cossart, 2012). Septins are highly conserved in vertebrates, and in humans are categorised into four groups (called the SEPT2, SEPT3, SEPT6 and SEPT7 groups), the products of which assemble into hetero-oligomeric complexes, filaments and ring-like structures. Septins can recognise areas of micron-scale curvature, including the cytokinetic furrow (see Glossary, Box 1) and phagocytic cup (see Glossary, Box 1), where they act both as scaffolds for protein recruitment and as diffusion barriers for subcellular compartmentalisation (Saarikangas and Barral, 2011; Mostowy and Cossart, 2012; Bezanilla et al., 2015; Cannon et al., 2017). Consistent with this, new work has shown that septins in neurons contribute to cell shape memory (Boubakar et al., 2017) and the maturation of dendritic spines (Yadav et al., 2017).

During *Shigella* infection, septins are recruited to the phagocytic cup alongside actin and form rings around invading bacterium (Mostowy et al., 2009a,b). Although the precise role of septins during bacterial entry is not clear, the depletion of SEPT2 by small interfering (si)RNA significantly reduces *Shigella* entry into host cells (Mostowy et al., 2009a). Following bacterial escape from the phagosome to the cytosol, septins are recruited to actin-polymerising bacteria, forming cage-like structures around *Shigella* that inhibit cell-cell spread, in a process called septin caging (Mostowy et al., 2010). The depletion of SEPT2, SEPT9 or nonmuscle myosin II (see Glossary, Box 1) inhibits septin caging and increases the number of bacteria with actin tails, whereas increasing SEPT2-nonmuscle myosin II interactions using tumour necrosis factor (TNF; see Glossary, Box 1) increases septin caging and prevents the formation of actin tails (Mostowy et al. 2010). Importantly, septin cages have been observed in zebrafish cells *in vitro*, as well as *in vivo* (Fig. 2C), supporting their role as an evolutionarily conserved host defence assembly (Mostowy et al., 2013).

The investigation of septins *in vivo* using mouse models has been challenging, considering that their deletion can often result in embryonic lethality (reviewed in Kinoshita and Noda, 2001; Mostowy and Cossart, 2012). However, developmental studies using zebrafish have linked the depletion of some septins (i.e. Sept6, Sept9a, Sept9b, Sept15) to growth defects and aberrant left-right asymmetry (Landsverk et al., 2010; Dash et al., 2014, 2016; Zhai et al., 2014). Zebrafish septins have also been shown to play a crucial role during *S. flexneri* infection (Mazon-Moya et al., 2017). In this case, depletion of Sept15 or Sept7b [zebrafish orthologues (see Glossary, Box 1) of human SEPT7] significantly increases host susceptibility both to compartmentalised (HBV) and systemic (caudal vein) *Shigella* infection. Live-cell imaging of Sept15-depleted larvae during infection revealed a failure of neutrophils to control infection. Susceptibility of septin morphants (see Glossary, Box 1) to infection is strictly dependent on the *Shigella* T3SS, as infection with T3SS-deficient *S. flexneri* is equally controlled by control or Sept15 morphants (Mazon-Moya et al., 2017). Sept15 morphants also exhibit neutropenia (see Glossary, Box 1; Mazon-Moya et al., 2017), a marker of poor prognosis of infection in humans. These data are consistent with experiments using Sept7-deficient transgenic mice showing impaired granulopoietic potential of myeloid progenitors (Menon et al., 2014). The use of morpholinos to target interferon regulatory factor 8 (Irf8; see Glossary, Box 1) and boost neutrophil numbers in Sept15 morphants failed to improve the outcome from *Shigella* infection, suggesting a cell-autonomous defect of neutrophils with nonfunctional Sept15 (Mazon-Moya et al., 2017). Collectively, studies of zebrafish infection with *Shigella* illustrate a new role for septins in host defence against bacterial infection, and highlight the crucial role of the cytoskeleton in neutrophil development and behaviour.

### Cellular immunity to *Shigella* infection

*Shigella* has emerged as a model pathogen to investigate how bacteria can be recognised by, or escape from, the host immune system (reviewed in Phalipon and Sansonetti, 2007; Ashida et al., 2011, 2015). For example, studies using *Shigella* have helped to discover major roles for nucleotide-binding oligomerisation domain (NOD)-like receptors (NLRs) (Girardin et al., 2003), bacterial autophagy (Ogawa et al., 2005) and inflammasomes (see Glossary, Box 1; Willingham et al., 2007) in host defence. This section will discuss cellular immunity to *Shigella*, and how this knowledge has evolved following recent *in vivo* studies using zebrafish.

### *Shigella*-autophagy interactions

Discovered as a nutrient recycling process in response to starvation (reviewed in Yang and Klionsky, 2010), autophagy is also a selective process crucial for cellular immunity (reviewed in Levine et al., 2011). During *Shigella* infection, cytosolic bacteria are recognised by the autophagy machinery and targeted for lysosomal delivery (Ogawa et al., 2005). The cytoskeleton, well known for its role in bacterial invasion and motility, also plays an important role during the autophagy response to bacteria (reviewed in Mostowy, 2014; Mostowy and Shenoy, 2015). Work using human epithelial cells has shown that septin cages colocalise with ubiquitylated proteins that concentrate around autophagy-targeted *Shigella* (Mostowy et al., 2010). Consistent with the notion that septin cage assembly and autophagy of bacteria are interdependent, the number of *Shigella* septin cages is significantly reduced following siRNA knockdown of autophagy machinery components (Mostowy et al., 2010; Mostowy et al., 2011). Moreover, *Shigella* mutants lacking IcsB, a T3SS effector involved in autophagy evasion, are compartmentalised into septin cages more efficiently than their wild-type counterparts (Mostowy et al., 2010; Mostowy et al., 2011). Importantly, ∼50% of *Shigella* entrapped in septin cages are metabolically inactive, highlighting septin caging as an antibacterial mechanism (Sirianni et al., 2016). In one of the first studies to use zebrafish for investigation of bacterial autophagy *in vivo* (Mostowy et al., 2013), infection of larvae with *S. flexneri* revealed that autophagy is a crucial component of innate immunity at the whole-organism level (Fig. 2D). In this case, depletion of the autophagy receptor protein p62 significantly reduced septin cage-associated *S. flexneri*, and increased bacterial burden and host mortality following infection. How septin cages assemble and interact with other cytoskeletal proteins during the autophagy response to bacteria, and their ability to be manipulated for therapy, is currently the focus of intense investigation.

Following T3SS-mediated invasion of epithelial cells, a number of host cell mechanisms recruit the autophagy machinery to *Shigella*, including the NLRs NOD1 and NOD2 that detect bacterial peptidoglycan (see Glossary, Box 1) in the cytosol (reviewed in Krokowski and Mostowy, 2016; Keestra-Gounder and Tsolis, 2017). NOD1/2 receptors can recruit ATG16L1, a protein crucial for autophagosome (see Glossary, Box 1) development, and inhibit the release of proinflammatory cytokines (Sorbara et al., 2013). A separate autophagy pathway follows phagocytic rupture, in which the autophagy machinery is recruited to damaged phagosomal membranes labelled by ubiquitin (Dupont et al., 2009; Thurston et al., 2012). In the cytosol, ATG5 interacts with the bacterial IcsA and targets *Shigella* towards autophagy and septin caging (Ogawa et al., 2005; Mostowy et al., 2010). A role for mitochondria in the promotion of septin cage assembly and bacterial autophagy has also been demonstrated (Sirianni et al., 2016). To avoid autophagic destruction, actin-polymerising *S. flexneri* can fragment mitochondria to counteract the assembly of septin cages (Sirianni et al., 2016). Rapamycin, a pharmacological inhibitor of mammalian target of rapamycin (mTOR; see Glossary, Box 1), stimulates autophagy and has been proposed as a therapy to promote bacterial clearance (Abdel-Nour et al., 2014). Indeed, *in vitro* experimental work in a variety of cultured cell models has shown that rapamycin can enhance the killing of bacteria (Gutierrez et al., 2004; Tattoli et al., 2012). However, rapamycin treatment of *S. flexneri*-infected zebrafish failed to rescue the infected hosts (Mostowy et al., 2013), suggesting that therapeutic manipulation of autophagy *in vivo* is complex. Similar results were observed following rapamycin treatment of *M. marinum*-infected zebrafish, which also failed to rescue the infected hosts (van der Vaart et al., 2014).

### *Shigella*-inflammasome interactions

In human (Zychlinsky et al., 1992) and zebrafish (Mostowy et al., 2013) macrophages, *Shigella* infection induces cell death, yet the underlying mechanisms are not fully understood. Interestingly, targeted ablation of nascent macrophages in transgenic zebrafish larvae Tg(*mpeg1*:Gal4-FF)^gl25^/Tg(UASE1b:*nfsB*.mCherry)^c26^ using metronidazole (see Glossary, Box 1) decreased larval survival following *S. flexneri* infection (Mazon-Moya et al., 2017), indicating that macrophages can somehow protect against infection *in vivo.* Experimental work performed *in vitro* using cultured cell models has shown that *Shigella* can manipulate inflammasome activity (reviewed in Hermansson et al., 2016; Suzuki et al., 2017). Inflammasome-triggered cleavage of interleukin (IL)-1β and IL-18 via cysteine-aspartic protease (caspase)-1 (see Glossary, Box 1) recruitment results in pyroptosis (see Glossary, Box 1; Miao et al., 2011). A variety of exciting papers have used zebrafish to study inflammasome activation *in vivo*. For example, clearance of *S.* Typhimurium from zebrafish by neutrophils requires the interferon gamma (IFNγ)-inducible GTPase guanylate-binding protein-4 (GBP-4; see Glossary, Box 1) and the inflammasome-dependent production of prostaglandin D2 (see Glossary, Box 1; Tyrkalska et al., 2016). In a separate study, zebrafish infection with a strain of *Listeria monocytogenes* ectopically expressing flagellin, the principal component of bacterial flagella, was shown to activate the inflammasome in macrophages and decrease infection (Vincent et al., 2016). Although tools to study the inflammasome in zebrafish remain limited compared to mice, zebrafish can now be used to follow the assembly of apoptosis-associated speck-like protein containing a caspase-recruitment domain (ASC) specks (see Glossary, Box 1) *in vivo* (Kuri et al., 2017). It will next be of great interest to study the events and pathways underlying inflammasome activation and cell death during *Shigella* infection of zebrafish.

The host cytoskeletal components drive activation of the inflammasome and cell-autonomous immunity (see Glossary, Box 1; reviewed in Mostowy and Shenoy, 2015). Work has linked actin to inflammation control through regulation of the NACHT, LRR and PYD domain-containing protein 3 (NLRP3) (Pelegrin and Surprenant, 2009; Jin et al., 2013; Johnson et al., 2013) and pyrin inflammasome complexes (Kim et al., 2015; Standing et al., 2017). Actin polymerisation proteins can regulate the NLRP3 inflammasome by interacting with caspase-1 and other inflammasome components (Pelegrin and Surprenant, 2009; Jin et al., 2013; Johnson et al., 2013). Actin depolymerisation, as a consequence of mutations in WD repeat-containing protein (WDR1), can activate the pyrin inflammasome (Kim et al., 2015; Standing et al., 2017). In the case of *S. flexneri* infection, disruption of WASp results in increased inflammasome activity and decreased bacterial clearance (Lee et al., 2017)*.* Recent work using the *Shigella*-zebrafish infection model has discovered a new role for septins in inflammation control. Experiments in this study showed that Sept15 morphants exhibit significantly increased caspase-1 activity and cell death, indicating that septins restrict inflammation *in vivo* (Mazon-Moya et al., 2017). Considering that increased inflammasome activity results in increased levels of IL-1β (reviewed in Malik and Kanneganti, 2017), blocking IL-1β signalling represents an attractive therapeutic solution to reduce inflammation. Indeed, inflammation in *Shigella-*infected Sept15 zebrafish morphants can be dampened by treatment with anakinra, an IL-1 receptor antagonist used to treat inflammatory disorders in humans, which ultimately rescues neutrophil and host survival (Mazon-Moya et al., 2017).

Together, these examples illustrate that zebrafish larvae are well suited to study the breadth of cellular immune responses available to control *Shigella* infection and inflammation. Moving forward, it can be expected that studies using zebrafish will continue to identify the molecular determinants and events underlying cellular immunity to *Shigella* infection *in vivo*.

## New ways to control *Shigella* infection

The *Shigella*-zebrafish infection model is highly versatile, and can be used to illuminate novel research aiming to control bacterial infection. In this section, we discuss how infection of zebrafish can be used to investigate new concepts underlying infection control, including emergency granulopoiesis and the use of predatory bacteria to combat antimicrobial resistance.

### Emergency granulopoiesis

How infection and inflammation can mediate emergency granulopoiesis is a subject that is difficult to fully investigate *in vitro*. The zebrafish model, however, has recently provided valuable insights into the fundamental aspects of stem cell biology and host defence. Haematopoiesis is the hierarchical process by which haematopoietic stem and progenitor cells (HSPCs; see Glossary, Box 1) produce mature blood cells (King and Goodell, 2011; Jagannathan-Bogdan and Zon, 2013; Laurenti and Göttgens, 2018). Following bacterial infection, circulating populations of neutrophils can become exhausted. Although emergency granulopoiesis can replenish the exhausted neutrophils, the underlying molecular events are mostly unknown (Manz and Boettcher, 2014; Teng et al., 2017). In the zebrafish, emergency granulopoiesis takes place in the aorta-gonad-mesonephros (AGM) and caudal hematopoietic tissue (CHT), and is supplied by the proliferation and differentiation of HSPCs (Fig. 1). Strikingly, a zebrafish model of *S.* Typhimurium infection revealed a direct expansion of the HSPC niche in response to bacterial infection (Hall et al., 2012). The HSPC expansion arises by release of granulopoietic cytokines, including granulocyte colony-stimulating factor (GCSF; CSF3) (Stachura et al., 2013). Neutrophil expansion in the AGM and CHT is dependent on inducible nitric oxide synthase (iNOS) and the transcription factor CCAAT/enhancer-binding protein β (C/EBPβ) (Hall et al., 2012). Considering that *Shigella* induces inflammation and neutropenia during infection of zebrafish (Mazon-Moya et al., 2017), it will now be of great interest to use *Shigella* infection of zebrafish to further study HSPC signalling and emergency granulopoiesis, and their precise role in host defence. An in-depth understanding of the cellular and molecular mechanisms governing HSPC biology will be important to inform strategies for using the therapeutic manipulation of emergency granulopoiesis to treat bacterial infections in humans. As a natural programme of host defence, strategies to boost emergency granulopoiesis have great therapeutic potential; however, such treatments must balance immune stimulation with the risk of stem cell exhaustion.

### Predatory bacteria work alongside the host immune cells to treat *Shigella* infection

**Fig. 3.**
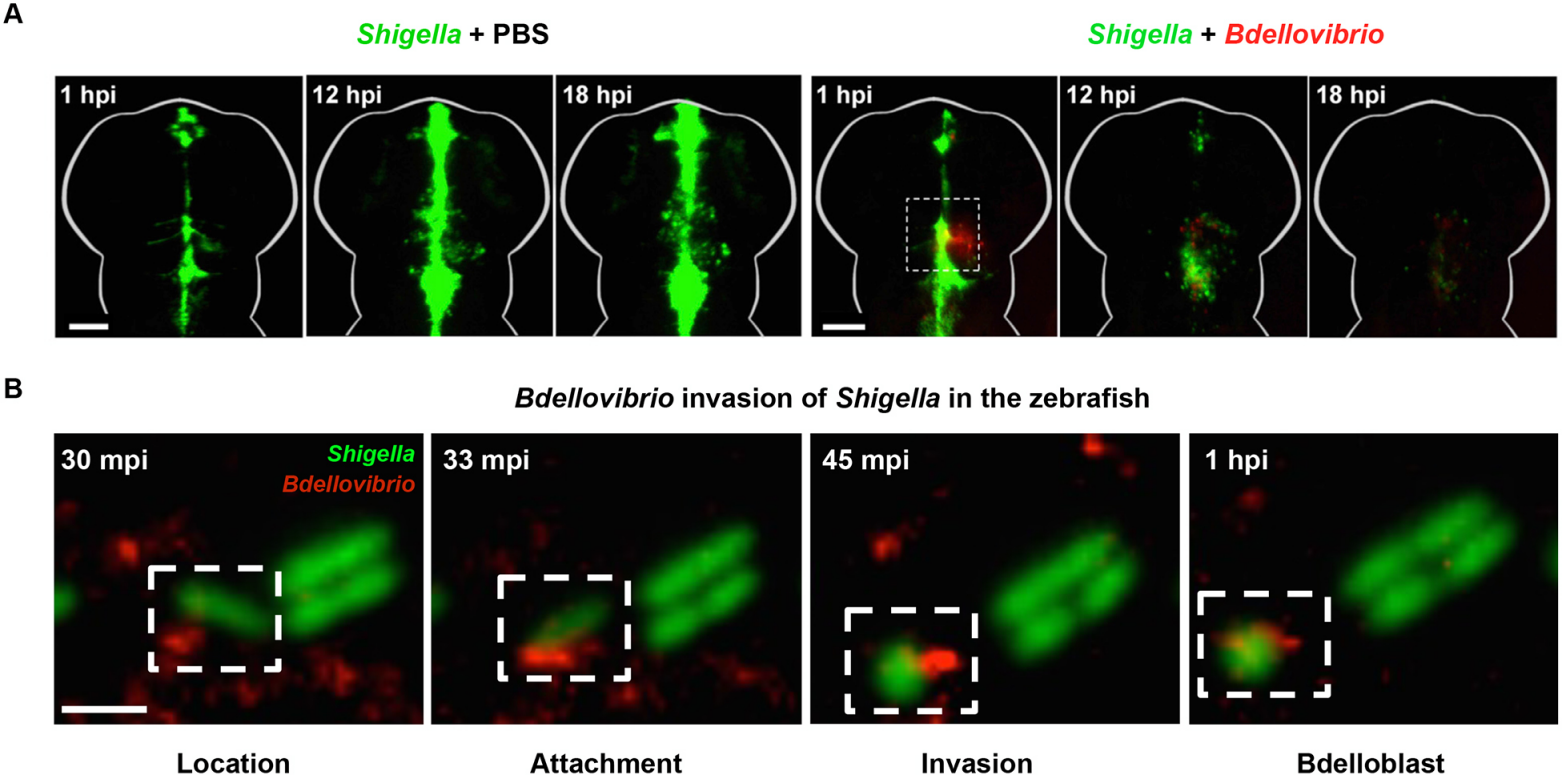
**Interaction of the predatory bacteria *Bdellovibrio* with *Shigella* in the zebrafish.** (A) Wild-type zebrafish larvae were injected in the hindbrain ventricle (HBV) at 3 days postfertilisation with >5×10^3^ colony forming units (CFUs) of GFP-*S. flexneri* (green), followed by hindbrain injection of either PBS or 1-2×10^5^ plaque forming units (PFUs) of mCherry-*Bdellovibrio* (red), 30-90 min after the initial *Shigella* infection. Representative images of the HBV in PBS- or *Bdellovibrio*-treated zebrafish larvae infected with *Shigella* are shown. The dashed line box shows the region of interaction between fluorescent *Bdellovibrio* and *Shigella*. For both treatments, the same larva was imaged over time. Scale bar: 100 µm. hpi, hours postinfection. (B) Representative images of *Shigella* predation by *Bdellovibrio in vivo* imaged by high-resolution confocal microscopy. Frames captured over time show stages of *Bdellovibrio* (red) invasive predation and rounding of *Shigella* (green). Scale bar: 2.5 µm. mpi, minutes postinfection. Adapted from Willis et al. (2016).

The emergence of antimicrobial resistant (AMR) bacteria is a global health threat, and improved antimicrobial therapies are urgently needed. The ESKAPE pathogens (*Enterococcus faecium*, *Staphylococcus aureus*, *Klebsiella pneumoniae*, *Acinetobacter baumannii*, *Pseudomonas aeruginosa* and *Enterobacter* species) are considered priority pathogens most likely to escape bactericidal treatment (Boucher et al., 2009), and are also recognised as new paradigms in microbial pathogenesis, transmission and resistance (Pendleton et al., 2013). An increased understanding of these pathogens can therefore lead to innovative strategies in the fight against antibiotic-resistant infections*. Bdellovibrio bacteriovorus* (see Glossary, Box 1) is a species of predatory bacteria gaining recognition for its ability to invade and kill Gram-negative pathogens (reviewed in Tyson and Sockett, 2017). *Bdellovibrio* have great potential to be used as ‘living antibiotics’ because they target pathogens using mechanisms that make acquiring resistance to predation difficult. Work has shown that *Bdellovibrio* can predate upon a wide variety of AMR Gram-negative bacteria *in vitro* (Kadouri et al., 2013). However, despite our understanding of bacterial predation *in vitro*, not much is known about the efficacy of bacterial predation *in vivo*. A recent study examined *Bdellovibrio* treatment following intranasal inoculation of rats with sublethal doses of *K. pneumoniae* (Shatzkes et al., 2016)*.* In this case, the authors found a 2-log reduction in *Klebsiella* burden in the lungs of the *Bdellovibrio*-treated rats. However, a follow-up study revealed that exposure of *Klebsiella*-infected rats to successive rounds of *Bdellovibrio* is ineffective against an acute blood infection (Shatzkes et al., 2017). One caveat of using mammalian models is that it is difficult to visualise the predator-prey interactions *in vivo*. To circumvent this, zebrafish larvae infected with multidrug resistant *Shigella* were used to study *Bdellovibrio* predation *in vivo* (Willis et al., 2016). This model system enabled, for the first time, the visualisation of bacterial predator-prey interactions *in vivo* in the presence or absence of an innate immune system (Fig. 3). In this study, immunocompromised larvae were generated using a morpholino targeting the transcription factor Pu.1 (Spi1b), which prevents myeloid cell differentiation. Control and Pu.1 morphants were injected with a lethal dose of *Shigella* in the HBV and treated with either phosphate buffered saline (PBS) or *Bdellovibrio* (Willis et al., 2016). Enumerations of *Shigella* from larval homogenates showed that treatment with *Bdellovibrio* reduced *Shigella* numbers in both immunocompromised and immunocompetent zebrafish larvae, but survival was significantly greater in the immunocompetent larvae. These experiments revealed that *Bdellovibrio* can work in synergy with the host immune cells (macrophages and neutrophils) to clear *Shigella* infection *in vivo*, before being cleared by the zebrafish immune system themselves. Next, an in-depth investigation of *Bdellovibrio* predation on a range of Gram-negative pathogens using the zebrafish model is needed before ultimately translating this procedure to higher vertebrates and humans.

## Conclusions and emerging perspectives

Here, we have illustrated how the zebrafish model can be used to study a variety of mechanisms underlying the host response to *Shigella* infection. The *Shigella*-zebrafish infection model is innovative because it is applicable to the *in vivo* milieu and generalisable across infectious agents, thus facilitating the comparison of evolutionarily distinct pathogens. In agreement with this, lessons learned from *Shigella* infection of zebrafish can be applied to other human bacterial pathogens (e.g. *Listeria*, mycobacteria, *Salmonella*), and also to neglected pathogens (e.g. *Burkholderia*, *Rickettsia*, *Waddlia*), of which little is known. Furthermore, *Shigella* infection of zebrafish can reveal general mechanisms of host defence relevant to combatting human infection from viral and fungal pathogens.

As an emerging animal model for the study of *Shigella* infection, the zebrafish offers numerous advantages and an exciting array of cell biology applications. However, there are some limitations to using the zebrafish model to study human bacterial infection. For example, there is a demand for improved tools, such as zebrafish-specific antibodies and knockout lines generated by CRISPR/Cas9. Another potential caveat is temperature: the optimal zebrafish growth temperature of 28°C mimics their natural environment, and deviations from this can affect their developmental cycle, whereas human pathogens are sometimes more virulent at 37°C, a temperature that in zebrafish can cause a depressed immune function and an upregulation of stress response proteins not present at 28°C (Long et al., 2012; Lam et al., 2013). Although zebrafish larvae offer the opportunity to study innate immunity in isolation, they cannot enable studies involving adaptive immunity. Experiments involving adaptive immunity will require the use of adult zebrafish, which have been used to study a variety of pathogenic bacteria, including *M. marinum* (Oehlers et al., 2015; Cronan et al., 2016) and *Mycobacterium leprae* (see Glossary, Box 1; Madigan et al., 2017). Lastly, the study of gene function in zebrafish can be challenging owing to the presence of paralogues (see Glossary, Box 1) within the genome (Howe et al., 2013). However, paralogues arising from duplication events in the genome can offer a redundancy, which can be beneficial for studies of genes for which knockout might otherwise result in embryonic lethality. Redundancy in the zebrafish genome has been beneficial to study a variety of genes crucial for host response to infection, including toll-like receptors (TLRs) (reviewed in Kanwal et al., 2014), NLRs (reviewed in Howe et al., 2016), and two Cxcl12 paralogues functioning as chemokines to activate phagocytes (Boldajipour et al., 2008).

As the *Shigella*-zebrafish model gains traction for the investigation of infection and cellular microbiology *in vivo*, a number of inspiring future directions are emerging (Box 2). Using this model, it will be of great interest to perform global gene expression profiling during infection for the discovery of new mechanisms underlying host defence, and to screen pharmacological compounds for therapeutic advance. To illuminate host-pathogen interactions, several interesting transcriptomic (Stockhammer et al., 2009; Benard et al., 2016) and proteomic (Díaz-Pascual et al., 2017) profiling studies have been used in zebrafish infected with bacterial pathogens; similar approaches can be used in zebrafish infected with *Shigella*. Moreover, ‘omics’ of isolated cells (e.g. macrophages or neutrophils) infected with *Shigella* can be illuminating to decipher bacterial virulence factors and host defence mechanisms important during this interaction. Although *Shigella* infection of adult zebrafish has not yet been tested, we propose that adult zebrafish could be used to study the adaptive immune response to *Shigella*, which is crucial to understanding the crosstalk between *Shigella* and T lymphocytes (reviewed in Salgado-Pabón et al., 2014) or to developing vaccine strategies (reviewed in Phalipon et al., 2008; Mani et al., 2016).

**Box 2. Outstanding questions in the field:**

- *Shigella* infection of zebrafish is valuable to discover novel mechanisms of cellular immunity. How generalisable are the lessons learned from *Shigella* infection of zebrafish to other bacterial pathogens?
- Can studies in zebrafish help to reveal where *Shigella* resides *in natura* outside the human host? Can zebrafish be humanised to emulate a more natural route of *Shigella* infection?
- What is the precise role of the cytoskeleton rearrangements (such as actin tails and septin caging) during *Shigella* infection *in vivo*? Can we manipulate the cytoskeleton during infection for a therapeutic benefit?
- Big data approaches (i.e. RNA sequencing, bacterial mutant libraries) are useful to discover new host pathways and/or bacterial effectors underlying infection. What can these approaches reveal when applied to *Shigella* infection of zebrafish?
- Can the *Shigella*-zebrafish infection experiments be used to inform experiments using higher vertebrate animal models and vice versa?

Recent work using mixed bacterial cultures *in vitro* and *in vivo* using rabbits and mice has shown that *S. sonnei* use a T6SS to outcompete *S. flexneri* (Anderson et al., 2017). These results highlight the T6SS as a key factor underlying the increasing prevalence of *S. sonnei* in developing countries (Lima et al., 2015; Kotloff et al., 2017), and will likely inspire the use of *S. sonnei* for cellular microbiology studies using zebrafish. Originally discovered in *in vitro* studies using *Shigella* infection of neutrophils, the release of neutrophil extracellular traps (NETs; see Glossary, Box 1) is currently regarded as an important cellular immunity mechanism for controlling a variety of pathogens, including *Staphylococcus*, *Klebsiella*, *Aspergillus* and *Candida* (Brinkmann et al., 2004; Branzk et al., 2014; Storisteanu et al., 2017). It is tempting to speculate that zebrafish infection models will be valuable to fully dissect the role of NETs in host defence *in vivo*. Recent work has shown in human cells (Wandel et al., 2017) and mice (Li et al., 2017) that *Shigella* evades GBPs by releasing IpaH9.8, an E3 ubiquitin ligase (see Glossary, Box 1) that triggers GBP degradation. *Shigella* infection of zebrafish can therefore be used to study GBPs and their precise role in host defence *in vivo*.

The field of pathogenesis is evolving from focusing on the mechanics of infection *in vitro* using cultured cells towards studying the infection process *in vivo* using animal models. The crypt culture model, known as intestinal organoids, is also powerful to study host-pathogen interactions (Nigro et al., 2016) and the response of the epithelium to enteric pathogens (Nigro et al., 2014). Considering the versatility of zebrafish infection models, it can be predicted that our understanding of *Shigella* pathogenesis will greatly benefit from *in vivo* applications of genome editing (e.g. CRISPR/Cas9), high-resolution imaging techniques (e.g. high-resolution fluorescence microscopy and cryo-electron tomography), and automated high-throughput screening approaches. Finally, it will be crucial that advancements in infection and cell biology learned from *Shigella* infection of zebrafish be applied for further study to higher vertebrates, including humans, and also as a therapeutic avenue in both human and veterinary medicine.
